# Recommendations for Establishing Therapeutic Alliance with Families of a person with severe mental health difficulties

**DOI:** 10.1192/j.eurpsy.2026.12129

**Published:** 2026-07-31

**Authors:** S. Strkalj Ivezić, D. Bosnjak Kuharic, A. Jambrosic Sakoman, A. Papic, J.-E. Leppänen

**Affiliations:** 1 University Psychiatric Hospital Vrapce, Zagreb, Croatia; 2 Department for psychotic disorders- female; 3 Department for psychotic disorders- male, University Psychiatric Hospital Vrapce, Zagreb, Croatia; 4 the Finnish family association FinFami, Helsinki, Finland

## Abstract

**Introduction:**

Therapeutic alliances with both patients and their families have been shown to improve treatment outcomes. Despite strong evidence and clinical guidelines, these practices remain insufficiently implemented. Families often experience unmet needs for information, support, and involvement in care, while professionals face barriers related to paternalism, stigma, and lack of training in collaborative approaches.

**Objectives:**

This work aimed to present recommendations for developing a therapeutic alliance with families, highlighting the benefits of a triadic partnership between patients, families, and clinicians and address barriers and propose strategies for implementation in routine clinical practice.

**Methods:**

We reviewed evidence-based interventions and international recommendations on family involvement in care. The synthesis focused on psychoeducation, communication training, motivational interviewing, and shared decision-making, as well as structured family therapies and support models.

**Results:**

Evidence shows that family interventions reduce relapse rates, improve quality of life, and decrease hospitalisation. Patients report greater support and engagement in their treatment, while families benefit from reduced stress and risk of burnout. Effective strategies include: Early establishment of collaborative dialogue with patient consent, stepwise involvement of relatives according to patient preferences, psychoeducation on illness and coping strategies, structured decision-making processes that respect patient autonomy and integrate family perspectives, training clinicians in empathy, active listening, and skills for managing transference and highly expressed emotion.

**Conclusions:**

A therapeutic alliance with families is a crucial recovery factor when indicated. It strengthens patient engagement, prevents relapse, and supports caregiver well-being. The implementation of structured, evidence-based family interventions should become a routine practice in mental health services. Developing professional skills and institutional support for collaboration with families is crucial to overcoming barriers and implementing recommendation into sustainable clinical outcomes.

**Disclosure of Interest:**

None Declared

